# 

**Published:** 1904-06-25

**Authors:** 


### NOTICE TO CORRESPONDENTS.

All MSS., Letters, Books for Review, and other matters intended for the Editor should be addressed The Editor, "The Hospital," The Hospital Buildings, 28 & 29 Southampton Street, Strand, W.C.

The Editor cannot undertake to return rejected MS., even when accompanied by stamped directed envelope.

**Notice to Advertisers and Subscribers.**

All Advertisements, Orders for Copies of The Hospital, and Business Communications should be addressed to **THE MANAGER (Not to the Editor),** The Hospital, 28 & 29 Southampton Street, Strand, London, W.C.

The Cost of Subscription, post free, is as follows:—Per week, 3½d.; per quarter, 3s. 3d.; per half-year, 6s. 6d.; per annum, 13s. Foreign and Colonial:—Per annum, 19s. 6d., payable, in all cases, in advance.

**NOTICE.—Vol. XXXV. of "The Hospital" (October 1903 to March 1904), in handsome cloth binding, is now on sale at the office of this Journal, price 10s. 6d. Cases for binding the Numbers contained in this Volume 2s. Index to Vol. XXXV., 3½d., post free.**

**The Monthly part for July will be ready on July 1. Price 1s., by post, 1s. 3d.**

### BOOKS RECEIVED.

Scientific Press, Limited.

"How to Become a Certified Midwife." By Dr. Appel.

Baillière, Tindall and Cox.

"The Diminishing Birth-rate." By J. W. Taylor.

Medical Monograph Series No. 10: "Cleft Palate and Hare-Lip."

J. and A. Churchill.

"The Personality of the Physician." By A. T. Schofield.

C. Griffin and Co., Limited.

"A Manual of Ambulance." By J. Scott Riddell.

J. B. Lippincott Co.

"International Clinics." No. 1, 14th Series.

Woodford, Fawcett and Co.

"A Modern Bee Farm." By S. Simmins.

Sherratt and Hughes, Manchester.

"Practical Prescribing and Dispensing." By W. Kirkby.

T. Fisher Unwin.

"'Liza of Lambeth." By W. Somerset Maugham.

### Medical Appointments.

**G. A. Barrow**, M.R.C.S., L.R.C.P.Lond., Honorary Anæsthetist to the Manchester Northern Hospital for Women and Children. **Harold Barwell**, M.B.Lond., F.R.C.S.Eng., Surgeon Laryngologist to the Mount Vernon Hospital for Consumption. **H. E. Davies**, L.R.C.P., M.R.C.S., Certifying Factory Surgeon for the Lutterworth District, Leicestershire. **W. McAdam, Eccles**, M.S.Lond., F.R.C.S.Eng., Examiner in Anatomy for the Fellowship of the Royal College of Surgeons of England. **W. M. Feldman**, M.R.C.S., L.R.C.P., Surgeon to s.s. *Merion* (American Line). **R. B. Fletcher**, M.B., Ch.B.Vict., reappointed Senior Resident Medical Officer at the St. Mary's Hospital for Women and Children, Manchester. **A. F. Heaton**, L.R.C.P., M.R.C.S. District Medical Officer of the Malmesbury Union. **S. Herbert Mason**, M.R.C.S., L.R.C.P.Lond., Honorary Anæsthetist to the Manchester Northern Hospital. **Sydney Scott**, B.S.Lond., F.R.C.S.Eng., Chief Assistant in the Aural Department, St. Bartholomew's Hospital, London, and Surgeon to Out-patients, Evelina Hospital for Sick Children, London. **W. H. Willcox**, M.D., B.Sc.Lond., D.P.H., Medical Registrar to St. Mary's Hospital.

### "The Hospital" Institutional Library.

"Hospitals and Asylums of the World." 4 Vols., with a Portfolio of Plans, complete £12 12s.

"Burdett's Hospitals and Charities, 1904." 5s. net.

"Burdett's Official Nursing Directory." 8s. net.

"Cottage Hospitals, General, Fever, and Convalescent." 10s. 6d.

"Uniform System of Accounts for Hospitals and Public Institutions." New Edition. 4s. net.

"Hospital Expenditure: The Commissariat." 2s. 6d.

"A Legal Handbook for the Use of Hospital Authorities." 2s. 6d.

"The Cottage Hospital Case Book or Register of Patients." 10s. and 12s. 6d.

All these are published by the Scientific Press, Ltd., and may be obtained through any bookseller, or direct from the publishers, 28 and 29 Southampton Street, Strand, London, W.C.

"Valuable little handbook."
*Nursing Notes.*

THE BURDETT SERIES.

"Can be strongly recommended to nurses."
*Treatment.*

### THE BURDETT SERIES OF POPULAR TEXT-BOOKS on NURSING.

Small Crown 8vo. Bound in cloth boards. Price **1/-** each post free; or the Series complete **10/-** post free.

**Each Volume contains about 100 pages written by a competent authority on the subject treated.**

No. 1.—**PRACTICAL HINTS ON DISTRICT NURSING.** By Amy Hughes.

No. 2.—**HOSPITAL AND PRIVATE NURSING.** By S. E. Orme.

No. 3.—**THE MIDWIVES' POCKET-BOOK. With Prefatory Chapter on the Midwives Bill.** By Honnor Morten, Author of "The Nurse's Dictionary," &c., &c.

No. 4.—**FEVERS AND INFECTIOUS DISEASES:** their Nursing and Practical Management. By William Harding, M.D.Ed., M.R.C.P.Lond., Author of "Mental Nursing," &c.

No. 5.—**NOTES ON PHARMACY AND DISPENSING FOR NURSES.** By C. J. S. Thompson.

No. 7.—**THE CARE OF CONSUMPTIVES.** By W. H. Daw, M.R.C.S., L.R.C.P.Lond.

No. 8.—**HOME NURSING OF SICK CHILDREN.** By J. D. E. Mortimer, M.B., F.R.C.S., L.S.A.

No. 9.—**MENTAL NURSING.** By William Harding, M.D.Ed., M.R.C.P.Lond.

No. 10.—**SICK NURSING AT HOME.** By Miss L. G. Moberly.

No. 11.—**GYNÆCOLOGICAL NURSING.** By G. A. Hawkins-Ambler, F.R.C.S., Author of "The Commonwealth of the Body."

No. 12.—**SYLLABUS OF LECTURES TO NURSES.** By Andrew Davidson, M.D.

*To be obtained of all Booksellers, or of*

The SCIENTIFIC PRESS, LTD., 28 & 29 Southampton Street, Strand, London, W.C.

